# A Decade of Research at the Intersection of Additive Manufacturing and Wearable Technology: A Bibliometric Analysis (2015–2025)

**DOI:** 10.3390/bios16030172

**Published:** 2026-03-20

**Authors:** H. Kursat Celik, Samet Şahin, Allan E. W. Rennie, Nuri Caglayan, Ibrahim Akinci

**Affiliations:** 1 Department of Agricultural Machinery and Technology Engineering, Akdeniz University, Antalya 07070, Türkiye; nuricaglayan@akdeniz.edu.tr (N.C.); iakinci@akdeniz.edu.tr (I.A.); 2 School of Engineering, Lancaster University, Lancaster LA1 4YW, UK; s.sahin@lancaster.ac.uk (S.Ş.); a.rennie@lancaster.ac.uk (A.E.W.R.)

**Keywords:** additive manufacturing, wearable technologies, bibliometric analysis, science mapping, bibliometrix

## Abstract

Additive Manufacturing (AM) and Wearable Technologies (WT) have rapidly evolved over the past decade. AM offers highly customisable fabrication, while WT enables minimally invasive health monitoring. The intersection of these fields presents emerging opportunities in biomedical and engineering domains. This study aims to map the scientific landscape of AM–WT research between 2015 and 2025 through a comprehensive bibliometric analysis. A total of 718 peer-reviewed publications were extracted from Web of Science (WoS), Scopus, and PubMed, following PRISMA-ScR guidelines. Using RStudio and the Bibliometrix package, analyses included co-authorship, citation trends, keyword co-occurrence, and thematic mapping. Custom author disambiguation scripts enhanced data quality and reliability. An annual publication growth of 24.89% was observed, with notable increases after 2020. Core themes included 3D printing, biosensors, microfluidics, and organ-on-a-chip devices. A shift from manufacturing-oriented research to biomedical integration is evident. Research output is dominated by the US, China, and South Korea, with moderate but not yet highly internationalised collaboration. The field of AM–WT research is undergoing a decisive transition from fabrication-focused studies to interdisciplinary, application-driven innovations. This shift is marked by increasing integration in healthcare and bioelectronics, yet hindered by regional imbalances and thematic gaps. Addressing these will be critical to advancing global impact. This study offers a cross-database bibliometric overview of AM–WT research. By combining three major data sources, it provides enhanced coverage and introduces novel analytical dimensions to guide future interdisciplinary efforts in personalised healthcare and wearable device innovation.

## 1. Introduction

### 1.1. Background on Additive Manufacturing

Additive Manufacturing (AM) is an emerging technology (also known as 3D printing) and has seen substantial technological advancement since its invention. It started as a rapid prototyping innovation and has since developed into a mature manufacturing technology. The first light-curing resin-based 3D printing system, recorded by Hull et al. in 1984, heralded the beginning of a technological revolution in manufacturing [1,2]. Today, it allows for the development of complex shapes and customised products that cannot, or are very difficult to, manufacture using traditional subtractive manufacturing. This is of significance not only for manufacturing technology but also for various other fields, including the biological, textile, and electronics sectors [3]. The capability of the technology to manufacture customised, lightweight, and complex components of various structures is the main reason for its increasing use across various sectors [4]. Furthermore, it is notably efficient, iterative, and waste-reducing, which fits well with the increasing need for the sustainable manufacturing of customised products [5]. Each of these aspects of the technology creates a new platform for emerging applications that require integrating structure, mechanics, and functionality in a single device.

### 1.2. Rise of Wearable Technology/Bio-Integrated Devices

Alongside improvements in manufacturing, the wearable (or bio-integrated) technology (WT) industry has undergone remarkable maturation. Wearables began as proof-of-concept devices, then advanced toward complex systems for real-time physiological observation, biosensing, and partially implantable use. The path for wearables mirrors the increasing need for individually customised healthcare observation, shapes, and seamless integration with the body. For the biomedical field, wearables and, accordingly, implantable devices, enable the inception of a new paradigm for individually customised patient treatment, around-the-clock observation, and minimally invasive monitoring [6]. It must also be recognised that the growing use of biosensor wearables relies on material science and processing that can accommodate comfort, conformability, and integration, requirements that regular processing technology cannot readily accommodate [7]. Recent studies further demonstrate rapid advances in multifunctional wearable systems incorporating biocompatible polymers, hybrid nanocomposites, and advanced bio-macromolecular matrices for flexible sensing and therapeutic applications, underscoring the accelerating diversification of wearable fabrication strategies [8,9].

### 1.3. Technological Convergence Between AM and Wearable Systems

The intersection of AM and WT arises naturally from the complementary strengths of both domains. AM enables the fabrication of customised, geometry-complex, and flexible structures; WT demands exactly these features when interfacing with the dynamic human body. Recent studies demonstrate how 3D printing of polymer composites facilitates the production of wearable sensors and flexible electronics that are difficult to achieve through conventional manufacturing [10,11]. Moreover, microfluidic devices, essential for many next-generation wearable biosensors (e.g., sweat analysis, continuous biochemical monitoring), benefit significantly from AM. Compared to traditional photolithography or etching-based methods, 3D printing offers shorter fabrication cycles, greater design freedom, and lower costs [7]. This enables the development of on-skin or flexible multipurpose biosensors, combining structural, fluidic, and electronic functionalities in a unified device. Such convergence is already being realised in the recent literature: a 2025 synthesis emphasises the pivotal role of additive manufacturing in advancing wearable and implantable biosensors, enabling patient-specific care and real-time health management [6].

### 1.4. Current Research Landscape and Fragmentation

Despite growing interest in the AM–WT convergence, the research landscape remains fragmented and highly interdisciplinary. On one hand, there is a proliferation of studies on AM applied to biomedical devices, microfluidic systems, smart textiles, and wearable biosensors. For instance, comprehensive reviews document AM’s applications in biomedical devices broadly, highlighting both successes and technical challenges [3,4]. Conversely, within WT and smart textiles, there is still a strong division between fashion-oriented 3D printed garments and function-oriented biosensing or electronics. While some studies emphasise material innovation, printing methods, and textile integration, others focus on biomedical sensing, microfluidics, or electronic function. This diversity, while promising, leads to a dispersed body of literature lacking a systematic synthesis of overarching themes, collaborative networks, or temporal evolution. In other words, despite evident momentum, the field lacks an up-to-date comprehensive map of how different strands, structural design, materials science, electronics, and bioengineering interrelate and evolve.

### 1.5. Limitations of Existing Reviews and Knowledge Gaps

Several key reviews have addressed elements of this convergence. For example, a 2023 integrative overview of 3D and 4D printing in textiles identifies the current state of the art in smart-textile research, but notes that the field remains embryonic, with significant challenges in scalability and industrial viability [4]. Similarly, dedicated reviews of 3D-printed biomedical devices provide valuable snapshots of technological status, yet do not systematically analyse bibliometric patterns, such as how research output has evolved, which institutions or countries lead in this domain, or how thematic clusters have formed [3]. In the domain of wearable biosensors, though recent work (e.g., Maji et al., 2025) underlines the transformative potential of AM, these analyses remain conceptually narrow, often focusing on case studies or device-level performance rather than the structural evolution of the research field as a whole [6]. Additionally, over roughly the last decade, several narrative and systematic reviews have progressively mapped how AM underpins the development of WT, particularly in biomedical contexts.

Early syntheses in the period surveyed emphasised 3D-printed sensors for clinical and physiological monitoring more broadly, showing how AM enabled miniaturised, patient-specific biosensors, soft implantable devices and microfluidic platforms, with wearables discussed as one key application domain within this wider landscape [12,13]. More focused work has subsequently examined 3D printing specifically for wearable (bio)sensors, detailing how vat photopolymerisation, material extrusion and material jetting processes can directly pattern glucose, lactate, sweat, strain and tactile sensors onto flexible substrates, and highlighting design strategies for conformal, skin-integrated devices [14]. In parallel, energy-related reviews have explored 3D-printed wearable energy storage and energy harvesting devices, framing structural batteries, supercapacitors and triboelectric or piezoelectric harvesters as essential to self-powered wearables and stressing the trade-offs between printability, mechanical compliance and electrochemical performance [15,16].

Most recently, reviews centred explicitly on AM for wearable sensors and bioelectronics underline how emerging printing modalities (for example, multi-material direct ink writing and hybrid extrusion–inkjet systems) can democratise access to personalised, low-cost health monitoring, while also extending coverage from on-skin platforms to fully wearable and implantable biosensor architectures [6,17]. Collectively, these review articles confirm that the AM–WT interface has matured into a recognisable research area, but they typically remain technology- or function-specific; none provide a quantitative, science-mapping synthesis of the overall AM–WT research landscape across multiple databases over the 2015–2025 period, precisely the gap the present bibliometric study addresses. Thus, a systematic bibliometric review, encompassing multiple databases across a significant time span, is still lacking. Such a review would allow identification of publication trends, key authors and institutions, international collaboration networks, thematic evolution, and technological gaps.

### 1.6. Rationale for a Multi-Database Bibliometric Assessment

Given the multidisciplinary and rapidly evolving nature of AM–WT research, a bibliometric approach that integrates multiple databases (e.g., WoS, Scopus, PubMed) offers distinct advantages. Relying on a single source may bias the sample toward particular disciplines (e.g., materials science, biomedical engineering, fashion/textiles) and under-represent inter-disciplinary studies. By combining databases, a broader and more representative dataset can be captured, including smart textiles, biomedical devices, biosensors, and microfluidics, thus enabling a more comprehensive mapping of the field. Bibliometric analysis allows for systematic de-duplication, author name disambiguation, and network analysis (co-authorship, co-citation, keyword co-occurrence), thereby revealing the structural and intellectual backbone of the domain. This methodology will reveal where the research is concentrated and how it evolves over time, which themes emerge, which fade, and where future opportunities may lie.

Online bibliographic databases function as critical archives of structured metadata for academic publications and serve as primary data sources for the quantitative mapping of scientific research. Within this context, WoS, Scopus, and PubMed are distinguished by their comprehensive subject coverage and reliable citation indexing, rendering them especially useful for bibliometric studies of interdisciplinary and technologically converging fields such as AM and WT. WoS and Scopus are acknowledged for their extensive journal indexing, uniform metadata frameworks, and proven effectiveness in scientometric analysis [18,19,20,21]. Meanwhile, PubMed, curated by the U.S. National Library of Medicine, delivers authoritative coverage of biomedical and bioengineering literature, making it essential when investigating areas such as wearable biosensors, implantable devices, or bioanalytical microfluidic systems [22].

### 1.7. Aim and Contribution of This Study

This study aims to conduct a systematic bibliometric review of research at the intersection of AM–WT over the period 2015–2025, drawing on multiple major databases to ensure broad coverage. While previous bibliometric analyses have examined AM or WT independently, almost no study to date has systematically mapped their intersection as a convergent research domain using integrated multi-database evidence. By explicitly focusing on the AM–WT interface rather than parallel disciplinary trajectories, this work identifies co-evolving thematic clusters, structural collaboration dependencies, and cross-domain knowledge flows that remain invisible in single-field analyses. By doing so, it seeks to map the intellectual landscape, identify leading authors, institutions, and countries, delineate thematic clusters and research trajectories, and highlight emerging trends and gaps. In addition, this study contributes methodologically by applying rigorous data-integration and de-duplication procedures (e.g., DOI-based matching, author name disambiguation) to mitigate the fragmentation typical in multi-database reviews. The resulting “research map” will serve as a resource for engineers, biomedical researchers, material scientists, textile technologists and policy-makers interested in the development and application of customised, wearable and functional devices enabled by AM.

## 2. Methodology

### 2.1. Methodological Framework and Review Protocol

The methodological framework for this bibliometric analysis was designed to maximise comprehensiveness, minimise database-specific bias, and ensure the replicability of findings, principles central to rigorous scientometric research [23,24]. The review was conducted in accordance with the Preferred Reporting Items for Systematic Reviews and Meta-Analyses for Scoping Reviews (PRISMA-ScR) guidelines to ensure transparent documentation of evidence identification, screening, and inclusion [25,26]. Guidance from the Joanna Briggs Institute’s methodological framework for scoping reviews was incorporated to support the structured justification of eligibility boundaries in multi-database evidence mapping [27]. The analytical workflow adhered to the established science-mapping model, which organises bibliometric inquiry into five sequential phases: study design, data collection, data curation and harmonisation, data analysis, and data visualisation and interpretation [28].

### 2.2. Study Objective and Multi-Database Strategy

The primary objective of this study was to conduct a comprehensive bibliometric analysis to map the intellectual structure, thematic evolution, and collaborative networks within the interdisciplinary field of AM for WT over the decade from 2015 to 2025. A key strength of the methodology is its integration and merging of records from three complementary bibliographic databases. While bibliometric reviews in engineering and technology often rely solely on WoS and/or Scopus due to their robust citation indexing [20], this approach can overlook significant research at interdisciplinary frontiers. To construct a unified dataset that mitigates the coverage biases inherent in any single source, records from WoS, Scopus, and PubMed were systematically merged [29].

### 2.3. Search Strategy and Eligibility Criteria

A comprehensive electronic search strategy was deployed using a Boolean query across document titles and author keywords. The search logic conjoined AM-specific terminology *(“Additive Manufacturing”, “3D Printing”, “3D Bioprinting”, “Rapid Prototyping”, “Layered Manufacturing”, “Rapid Manufacturing”, “Rapid Tooling”, “Design for Additive Manufacturing”, “DfAM”)* with WT and bio-integrated device concepts *(“Wearable Device”, “Wearable Sensors”, “Implantable Devices”, “Implantable Sensors”, “Smart Sensors”, “Wearable Applications”, “Bioanalytical Devices”, “Wearable Microfluidics”, “Biosensors”, “Bioelectronics”, “Lab on a Chip”, “Organ on a Chip”, “Human body-on-a-Chip”, “Microfluidic Devices”)*. In this study, WT is operationally defined to include bio-integrated and body-proximal systems that function in direct physiological interface with the human body. Accordingly, implantable devices and organ- or lab-on-chip platforms were included when positioned within wearable or patient-specific monitoring architectures enabled by AM. This boundary reflects the increasing convergence of wearable, implantable, and microfluidic systems in translational bioengineering literature, rather than an expansion beyond the study’s conceptual scope. The search was deliberately restricted to titles and keywords, excluding abstracts, to maintain a precise focus on the core published contributions and to mitigate the risk of retrieving publications based on peripheral topics mentioned only in the abstract [30]. This design reflects a deliberate precision–recall trade-off common in bibliometric science mapping. While abstract-level searching may increase corpus size, it can substantially expand thematic heterogeneity by capturing studies where AM or WT are only tangentially referenced. By restricting the query to titles and author keywords, the analysis prioritises conceptual centrality and reduces false-positive inclusion. We acknowledge that this may exclude some peripheral or implicitly connected studies; however, it strengthens the structural validity of the mapped convergence domain. The search was confined to English-language research articles, review articles, and full-text conference papers published between 10 October 2015 and 10 October 2025. This deliberate selection criterion excluded non-peer-reviewed material, books, book chapters, editorials, conference abstracts, and letters, ensuring the analytical focus remained on the core, citable body of peer-reviewed scientific output, thereby enhancing the reliability and validity of the bibliometric indicators.

### 2.4. Data Integration and Curation Process

The data collection phase involved executing the refined search query across three major bibliographic databases: WoS-Core Collection, Scopus, and PubMed. The initial results were exported in the standard formats provided by each platform: plain text (.txt) files, including ‘Full Record and Cited References’, from WoS and PubMed, and a comma-separated values (.csv) file with ‘No Truncation’ from Scopus to ensure comprehensive metadata capture.

A fundamental and methodologically distinctive aspect of this study was the subsequent data integration and refinement pipeline, designed to overcome significant limitations inherent in standard bibliometric workflows. While established tools such as the bibliometrix R package [28] offer a native function to merge datasets by converting them into a proprietary “.Rdata” format, preliminary tests revealed a critical shortcoming for author-level analysis. The standard merging algorithm relies on the “surname + initial” (AU) field for author identification, which erroneously aggregates distinct authors who share the same surname and first initial (e.g., “Liu, Yuchao” and “Liu, Yu” both being standardised to “LIU Y”). This homonym problem poses a substantial threat to the validity of co-authorship network analysis and productivity rankings. To ensure a robust and accurate foundation, a custom, multi-stage data curation pipeline was implemented in the R environment [31] extending the core bibliometrix functions. The process began with the harmonisation of the three source files into the canonical bibliometrix data frame structure using the “convert2df()” function.

A hierarchical deduplication strategy was then applied, using a cleaned Digital Object Identifier (DOI) as the primary key, followed by a combination of normalised title and publication year for records lacking a DOI. Within each group of duplicates, the record with the highest metadata completeness score was retained, with priority given to WoS, followed by Scopus, and then PubMed, a strategy informed by cross-database coverage studies [32]. Critically, to resolve the author disambiguation issue, a custom R script was developed. This script leveraged the full author names (AF) available in the source metadata to generate unique, full-name-based author tokens (e.g., “Liu, Yuchao” → LIU_YUCHAO and “Liu, Yu” → LIU_YU). This tokenisation process effectively distinguishes between homonymous authors, ensuring that the subsequent merger and all author-level analyses are based on accurate, disambiguated entities. A separate lookup table was created to maintain transparency and traceability between the generated tokens and the original author names.

This rigorous process yielded a final, integrated corpus of 718 unique publications. The composition of the retained records was 436 from WoS, 279 from Scopus, and 3 from PubMed. A diagnostic assessment confirmed the high quality and fitness for bibliometric analysis. Critical fields such as Abstract (AB), Author (AU), Title (TI), and Publication Year (PY) demonstrated 100% completeness. Fields essential for citation and keyword analysis, including Cited References (CR; 99.44% complete), DOI (DI; 93.18% complete), and Author Keywords (DE; 93.31% complete), were all in a ‘Good’ or ‘Excellent’ state. The high DOI coverage and near-complete cited reference coverage ensure the robustness of subsequent citation-based analyses. The small but conceptually vital contribution from PubMed, which captured unique perspectives at the intersection of bioprinting and organoids [33], underscores the value of a multi-database approach in capturing emergent, interdisciplinary research lines that might be underrepresented in more focused, engineering-oriented databases. Here it should be underlined that although the numerical contribution of PubMed to the final corpus is limited (n = 3), its inclusion serves a methodological validation function. Specifically, cross-database querying allows assessment of coverage asymmetry and ensures that biomedical-facing AM–WT publications are not systematically excluded due to database indexing bias. The marginal quantitative yield, therefore, reflects overlap consolidation after DOI-based deduplication rather than redundancy of the biomedical search domain.

### 2.5. Data Analysis and Visualisation

**Figure 1:**
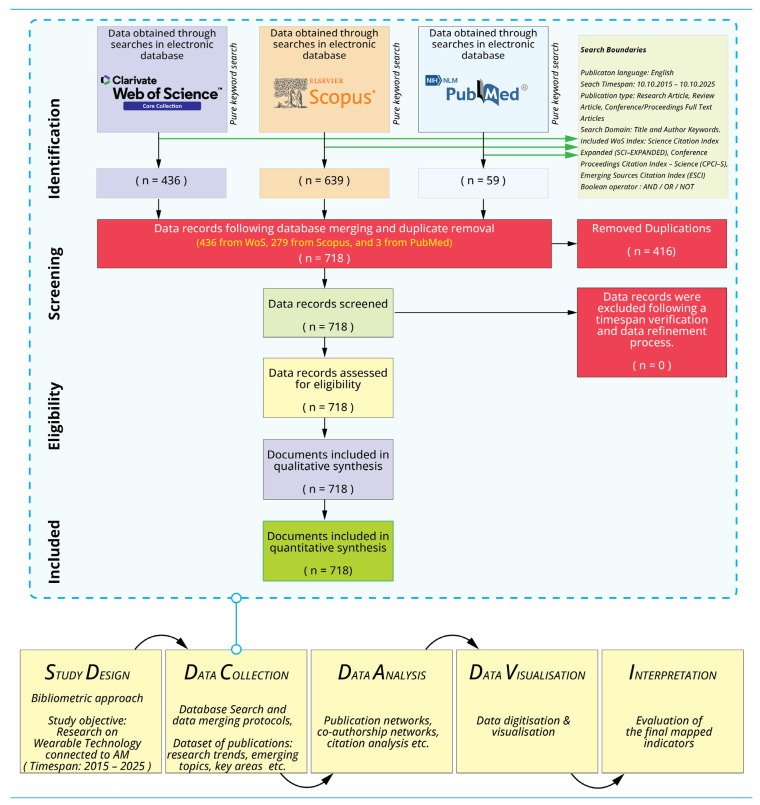
The mapping workflow and data collection protocol.

Data processing and analysis were conducted using the R programming language within the RStudio environment (V 2025.09.2 Build 418), leveraging the ‘Bibliometrix’ package (www.bibliometrix.org) [28]. This specialised toolset facilitated quantitative analysis of publication trends, construction of co-authorship and co-citation networks, and mapping of keyword co-occurrence and thematic evolution (Table 1). The complete data collection and screening protocol is delineated in Figure 1 (adapted from Page et al., 2021 [26] and Aria and Cuccurullo (2017) [28]).

## 3. Results and Discussion

### 3.1. Analysis Outputs and Interpretations

#### 3.1.1. Core Characteristics of the Analysed Publication Corpus

The publication corpus consists of 718 documents produced between 10 October 2015 and 10 October 2025, published across 315 journals and conference proceedings (Sources) (Table 2). The volume and spread of sources indicate a broad yet coherent research landscape. A sustained annual growth rate of 24.89%, together with an average document age of 4.52 years, shows that the field has expanded rapidly and is predominantly shaped by recent outputs. Citation indicators demonstrate moderate yet consistent scholarly influence. Each document has received an average of 29.33 citations, and the corpus collectively draws upon 29,275 references, reflecting both external visibility and a substantial underpinning knowledge base. The distribution of document types comprises 420 research articles, 167 review papers and 131 conference papers, which illustrates a balanced mixture of empirical work, conceptual consolidation and early-stage research. The authorship structure highlights a strong collaborative orientation. A total of 3496 authors contribute to the dataset, and the low number of single-authored papers (n = 12) contrasts with the overall average of 5.66 co-authors per publication. International collaboration is present in 15.46% of the outputs, pointing to a developing but not yet dominant cross-border research network. While this proportion reflects active cross-border engagement, it indicates that AM–WT research remains primarily embedded within national innovation systems. Given the interdisciplinary nature of the field, requiring advanced manufacturing infrastructure, materials science expertise, microfluidic engineering, and clinical translation, broader international integration may enhance knowledge transfer and accelerate standardisation across regulatory and technological environments. The interpretation of collaboration intensity should therefore be understood relative to the structural demands of convergence research rather than as an absolute benchmark against other engineering domains. Conceptual diversity is evident in the recorded keywords. The corpus includes 1971 Author’s Keywords and 3854 Keywords Plus, which together delineate both the explicit thematic choices of researchers and the wider intellectual patterns reflected in the cited literature. These indicators collectively portray a research domain characterised by steady growth, increasing thematic breadth and sustained collaborative activity.

#### 3.1.2. Annual Scientific Production and Citation Impact

**Figure 2:**
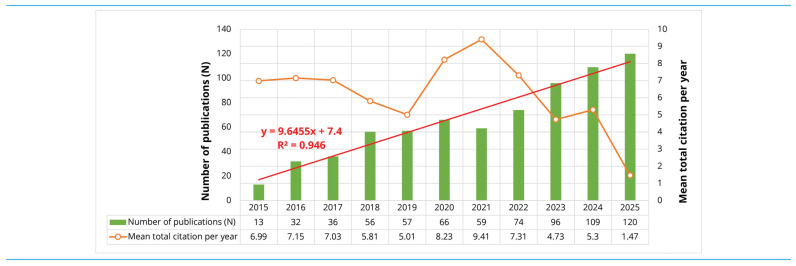
Annual scientific production and citation trends (2015–2025).

Annual publication output exhibits a clear and sustained upward trend, rising from 13 documents in 2015 to 120 in 2025, as shown in Figure 2. The underlying linear model (y = 9.6455x + 7.4, R^2^ = 0.946) indicates a highly predictable trajectory, with an average increase of roughly ten publications per year. This pattern reflects not only expanding research interest but also the progressive consolidation of AM and WT as an interdisciplinary research domain. Citation performance displays more variable temporal dynamics. Mean total citations per publication remain relatively stable between 2015 and 2017 (6.99–7.15) before declining moderately in 2018 and 2019 (5.81 and 5.01). The marked rise observed for the 2020 and 2021 cohorts (8.23 and 9.41) aligns with typical citation lifecycle behaviour, whereby publications benefit from both topical salience and sufficient time for scholarly uptake. The subsequent decrease from 2022 to 2025 (7.31, 4.73, 5.30 and 1.47) reflects citation latency effects rather than any substantive shift in research quality, because newer documents have had limited exposure for citation accrual. Taken together, the diverging trajectories of publication counts and short-term citation averages illustrate a field undergoing strong quantitative expansion while the most recent contributions are still progressing through the early stages of their citation lifecycle. The combination of steady output growth and temporally modulated citation performance is consistent with a rapidly developing research area in which visibility and scholarly integration continue to evolve.

#### 3.1.3. Thematic and Collaborative Structure: A Three-Field Analysis

**Figure 3:**
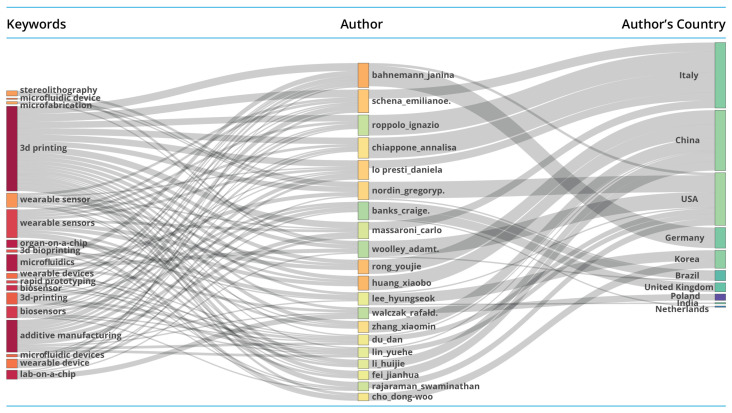
Three-field plot (Sankey diagram) illustrating the relationships between major research themes (**left**), prolific authors (**centre**), and their countries of affiliation (**right**). The thickness of the connecting bands represents the strength of association, indicating which authors are linked to specific research themes and how these are distributed across national research systems.

The three-field plot (Sankey diagram) in Figure 3 highlights a tightly integrated conceptual landscape centred on manufacturing technologies and their direct application to wearable and microfluidic systems. The dominant cluster, linking ‘3D printing’, ‘additive manufacturing’ and ‘stereolithography’ with ‘wearable sensors’, ‘wearable devices’ and ‘biosensors’, indicates that the field is structurally organised around fabrication techniques that enable functional device development. The presence of ‘microfluidic devices’, ‘lab-on-a-chip’, ‘organ-on-a-chip’ and ‘3D bioprinting’ as adjacent terms shows that biomedical microengineering has become an embedded and complementary research stream rather than a peripheral niche. The authors most closely associated with these themes, such as Zhang Xiaomin, Huang Xiaobo and Lin Yuehe, are predominantly affiliated with institutions in China, while key Italian contributors, including Roppolo, Chiappone and Massaroni, show similar thematic alignment. This concentration suggests that these national systems host research groups with sustained strategic focus on additive manufacturing and its microfluidic or wearable extensions. Secondary but notable contributions from the USA and Korea confirm broader international participation but with less pronounced thematic influence. The combined pattern indicates that intellectual development in the field is shaped by the interaction of three reinforcing factors: a coherent technological core, a small group of highly active authors and a limited number of national research hubs. The alignment between thematic centrality and geographic concentration suggests that institutional capacity and long-term programme-level commitments are key determinants of research direction, particularly in areas requiring advanced manufacturing and microfabrication capability.

#### 3.1.4. Field Maturity and Future Trajectory: A Life Cycle Analysis

**Figure 4:**
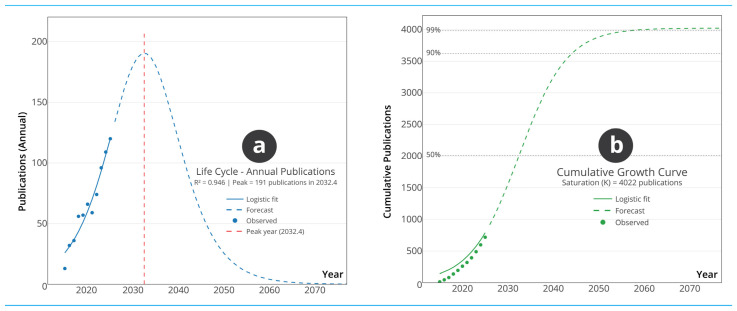
Logistic growth models of AM–WT research. (**a**) Annual publication model estimating the timing and magnitude of peak yearly output. (**b**) Cumulative publication model projecting the long-term saturation point of the field.

The logistic models in Figure 4 evaluate the developmental stage of research integrating AM with WT by examining both annual publication behaviour and long-term cumulative growth. The annual publication model in Figure 4a exhibits a strong fit to the logistic function (R^2^ = 0.941), indicating that the trajectory of yearly output follows a predictable growth pattern. The logistic formulation mentioned in this study follows the standard three-parameter logistic growth function $P(t)=\frac{K}{1+e^{-r(t-t_{0})}}$, commonly used to model bounded growth dynamics in innovation systems and scientometric life-cycle analyses [34]. In this expression, K represents the estimated carrying capacity, r the intrinsic growth rate, and $t_{0}$ the inflexion point corresponding to the maximum growth acceleration. Parameter estimation was conducted using non-linear least squares regression within the R environment. The modelling workflow was implemented by the Bibliometrix package within the data analysis [28], consistent with established bibliometric growth-modelling practices and methodological guidelines [24]. This formulation allows interpretation of publication dynamics within a bounded life-cycle framework rather than as an unconstrained linear extrapolation. The model estimates a peak production of approximately 191 publications around 2024. This projected peak, considered alongside the observed annual growth rate of 24.89%, suggests that the field is operating near the upper limit of its exponential growth phase. Such positioning typically reflects intense research activity, rapid thematic expansion and high responsiveness to emerging technological opportunities. The observed logistic behaviour suggests that the AM–WT interface is transitioning from rapid expansion to structured consolidation, indicating the emergence of stabilised research trajectories rather than transient exploratory activity. This inflexion implies that future innovation is likely to be driven less by exploratory proliferation and more by refinement, optimisation and application-level integration of established fabrication platforms. The cumulative publication model in Figure 4b estimates a saturation point of approximately 4022 documents. With 718 publications currently present in the corpus, only about 18% of the projected total output has been reached. This wide gap between current and expected cumulative volume demonstrates that the field has considerable future expansion potential. This estimate should be interpreted as a scenario derived from the continuation of current publication dynamics rather than as a deterministic forecast. Logistic growth modelling assumes structural continuity in research intensity, funding allocation, and technological momentum; disruptions such as paradigm-shifting breakthroughs, funding contractions, regulatory shifts, or saturation of key application domains may substantially alter this trajectory.

The young average document age of 4.52 years and the relatively high mean citation count further support the inference that the domain remains in a dynamic and formative phase, characterised by continuous methodological advancement and broadening application scope. Taken together, the models indicate that although annual output is approaching its predicted short-term peak, the field remains far from structural saturation. This combination of imminent peak activity and substantial long-term capacity suggests that research in AM connected to WT will continue to diversify and consolidate over the coming years. The life-cycle position, therefore, points to a domain that is both highly active in the present and poised for sustained development in the future. Comparable bibliometric life-cycle analyses in adjacent technological domains, such as additive manufacturing in general industrial contexts or wearable healthcare technologies independently, have demonstrated similar early-stage acceleration followed by structural consolidation once core platforms stabilise. The present projection should therefore be understood as consistent with observed maturation patterns in analogous innovation systems, while remaining sensitive to exogenous technological and policy drivers.

#### 3.1.5. Source Structure, Productivity and Influence

**Figure 5:**
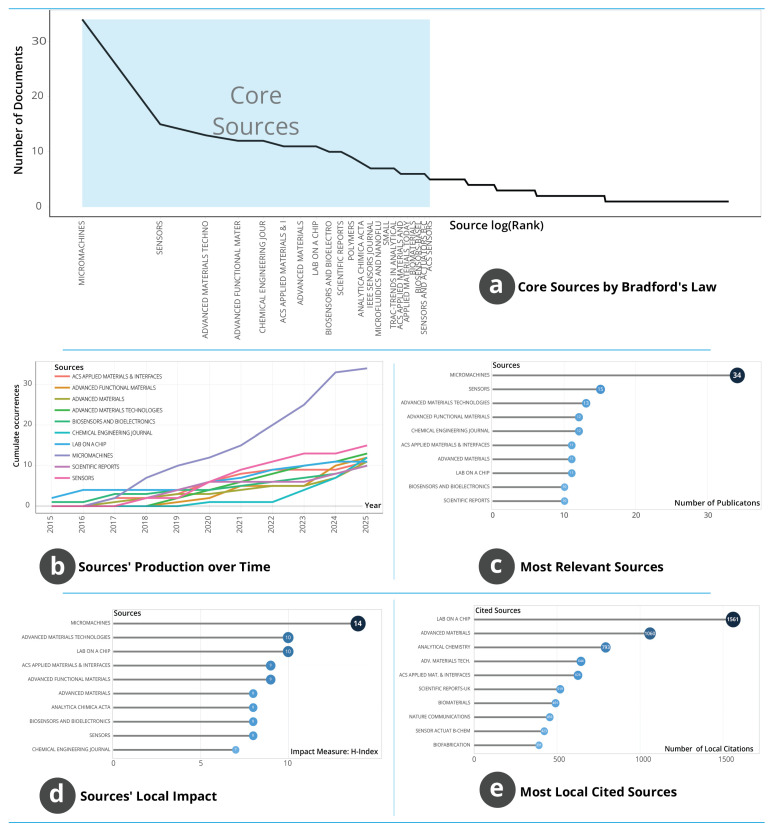
Source-level characteristics of AM–WT research: (**a**) Bradford distribution identifying core journals. (**b**) Cumulative publication trajectories of leading sources. (**c**) Most prolific journals by publication count. (**d**) Local H-index values indicating source impact. (**e**) Most locally cited journals within the corpus.

The source-level indicators shown in Figure 5 characterise how research in AM and WT is distributed, concentrated and cited across journals and conference outlets. The Bradford distribution in Figure 5a displays a steep initial decline followed by an extended tail, indicating that a small group of core journals accounts for a disproportionately large share of publications while many peripheral outlets contribute only occasionally. Journals such as *Micromachines, Sensors, Advanced Materials Technologies, Advanced Functional Materials* and *ACS Applied Materials & Interfaces* fall within this core, demonstrating their central role as primary dissemination platforms for methodological and device-oriented research within the field. Temporal patterns in Figure 5b show that most outlets maintained modest activity between 2015 and 2019, followed by marked increases from 2020 onwards. This acceleration is most prominent in *ACS Applied Materials & Interfaces, Advanced Materials* and *Advanced Materials Technologies*, each displaying steep cumulative growth in recent years.

In contrast, longstanding venues such as *Micromachines* and *Sensors* exhibit steadier trajectories, consistent with their established but specialised thematic focus. These patterns indicate a shift from early concentration in niche journals towards broader engagement by high-impact materials and engineering outlets, suggesting widening recognition of the field’s relevance. Figure 5c ranks journals by publication volume and confirms a hierarchy aligned with but not identical to the Bradford distribution. *Micromachines* and *Sensors* contribute the highest numbers of publications, followed by a mid-tier of outlets including *Advanced Materials Technologies*, *Advanced Functional Materials* and *Chemical Engineering Journal*. This distribution reflects both stable venues for microsystems and sensing research and the growing involvement of higher-impact multidisciplinary materials journals. Figure 5d assesses local influence through the H-index within the dataset. *Micromachines* again appears as the leading source, followed by *Advanced Materials Technologies* and *Lab on a Chip*. The ordering indicates that journals with high productivity also tend to record repeated citation use, but the pattern is not uniform across all venues. Consistency of citation performance in the dataset highlights the importance of a limited number of journals that function as recurring reference points for experimental and conceptual work.

The locally cited sources in Figure 5e reveal a different structure from both productivity and H-index patterns. *Lab on a Chip* receives the highest local citation count, followed by *Advanced Materials* and *Analytical Chemistry*. This suggests that the most influential conceptual and methodological foundations derive from journals specialising in microfluidics, analytical techniques and advanced materials, even when these outlets are not the most prolific. The divergence between productivity and influence underscores that high-impact methodological contributions may originate from journals with narrower but deeper conceptual reach. Overall, the source landscape is characterised by a small set of journals that dominate publication activity, complemented by a slightly broader group that anchors the field’s intellectual foundations. The combined evidence reflects a structurally mature yet expanding dissemination ecosystem that supports both exploratory and high-impact research.

#### 3.1.6. Author Structure, Influence and Productivity

**Figure 6:**
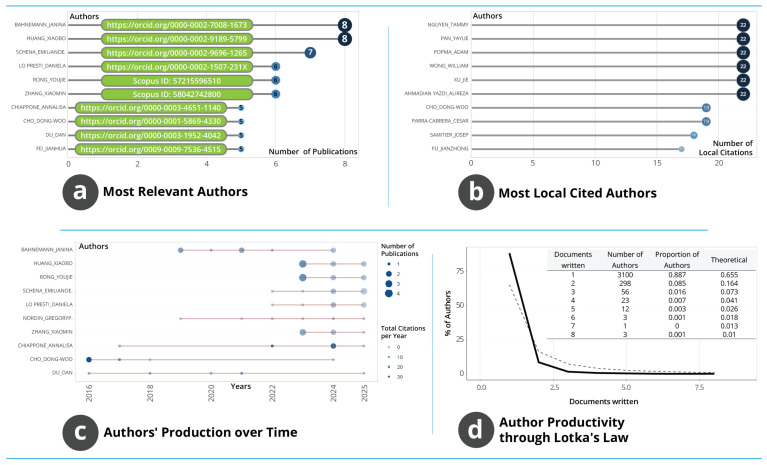
Author-level characteristics of AM–WT research. (**a**) Most prolific authors by publication count. (**b**) Authors with the highest local citation impact. (**c**) Temporal patterns of publication and citation activity. (**d**) Productivity distribution assessed against Lotka’s Law.

The authorship structure shown in Figure 6 illustrates how individual researchers contribute to and shape the field’s development. Figure 6a identifies the most prolific authors, with Bahnemann Janina and Huang Xiaobo each contributing eight publications, followed by Schena Emiliano with seven, and several researchers producing between five and six documents. This distribution indicates that while many scholars engage with the field, sustained high-frequency contribution is limited to a relatively small group whose continuous output supports thematic stability and methodological advancement. Figure 6b shows that author influence, measured through local citation counts, differs from productivity patterns. Researchers such as Nguyen Tammy, Pan Yayue, Popma Adam, Wong William and Xu Jie receive the highest local citation counts despite more modest publication volumes. This divergence demonstrates that conceptual influence and methodological impact are not solely determined by publication quantity; rather, key contributions arise from studies that introduce foundational techniques or frameworks that are repeatedly reused within the corpus.

Temporal patterns in Figure 6c show variability in publication timing and citation intensity across authors. Contributors such as Bahnemann, Huang, Rong and Schena display sustained activity from the late 2010s onwards, with notable increases after 2020. Others exhibit shorter but concentrated publication windows. The differing sizes of the citation circles indicate substantial variation in the visibility of individual outputs, shaped by both thematic relevance and proximity to periods of intensified research interest. These patterns confirm that the field is supported by overlapping cohorts of contributors whose participation aligns with technological developments and emerging application domains. Figure 6d evaluates productivity through Lotka’s Law. The empirical curve shows that nearly 89% of authors have a single publication, while only three authors have produced as many as eight publications. This steep decline from one-time to highly active contributors is characteristic of interdisciplinary research areas that attract wide but episodic participation while relying on a small core of consistently productive scholars.

The close alignment between empirical and theoretical Lotka distributions indicates that the authorship system follows expected scientometric regularities and reflects a structurally coherent research environment. Collectively, the evidence demonstrates a field shaped by a large peripheral group of occasional contributors and a small but influential core whose sustained productivity and methodological influence underpin the continuity and development of the research domain.

#### 3.1.7. Institutional Contributions

**Figure 7:**
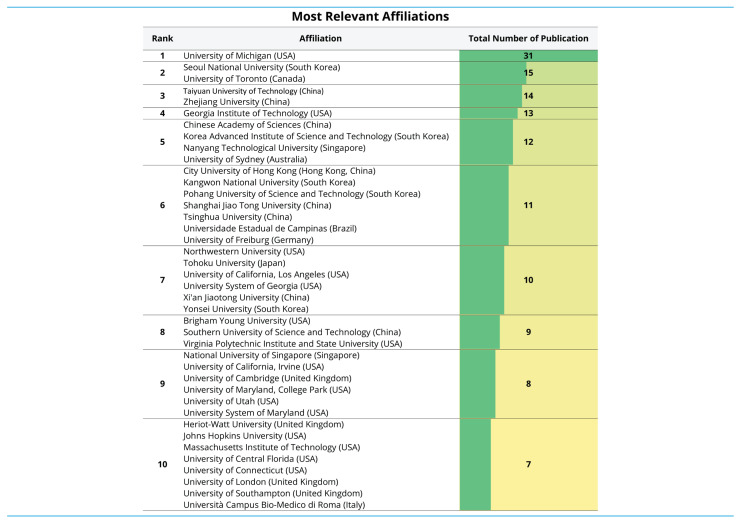
Institutional contributions to AM–WT research (system-level entities (multi-campus governance structures) and non-institutional platforms/consortia (e.g., national access platforms) were excluded to ensure attribution to identifiable research-performing institutions.

Figure 7 summarises the most productive institutions contributing to the AM-WT literature. The distribution indicates a clearly stratified pattern, in which a small number of institutions account for the highest outputs, followed by a broader set of organisations contributing at moderate but comparable levels. The leading contributor is the University of Michigan with 31 publications. A second tier is formed by Seoul National University and the University of Toronto (15 publications each). Subsequent ranks show closely clustered outputs, including Taiyuan University of Technology and Zhejiang University (14 publications), and Georgia Institute of Technology (13 publications). The remaining institutions in the top ranks contribute between 12 and 7 publications, highlighting a competitive and internationally distributed landscape spanning East Asia, North America, Europe, and Oceania. Notably, the presence of multiple institutions with identical totals within the same rank suggests that institutional productivity beyond the top position is characterised by near-parity among several globally active universities. To ensure interpretability at the level of individual research-performing institutions, the institutional list was compiled with explicit exclusion criteria applied to non-university and system-level records. First, umbrella entities representing multi-campus governance structures (e.g., “University System” or “System of Higher Education” labels) were excluded because they do not correspond to a single, clearly attributable university and may aggregate outputs from multiple campuses, thereby obscuring where research activity is actually located. In parallel, national access platforms and consortia (e.g., Egyptian Knowledge Bank) were not included because they do not represent employing academic institutions or research-performing organisations in the conventional affiliation sense; rather, such labels typically reflect dissemination, access, or administrative metadata and are not suitable for institutional productivity comparisons. These decisions improve the validity of institutional attribution and ensure that Figure 7 reflects contributions from identifiable universities and research-performing institutions.

#### 3.1.8. Geographical Distribution, Collaboration Patterns and Research Influence

**Figure 8:**
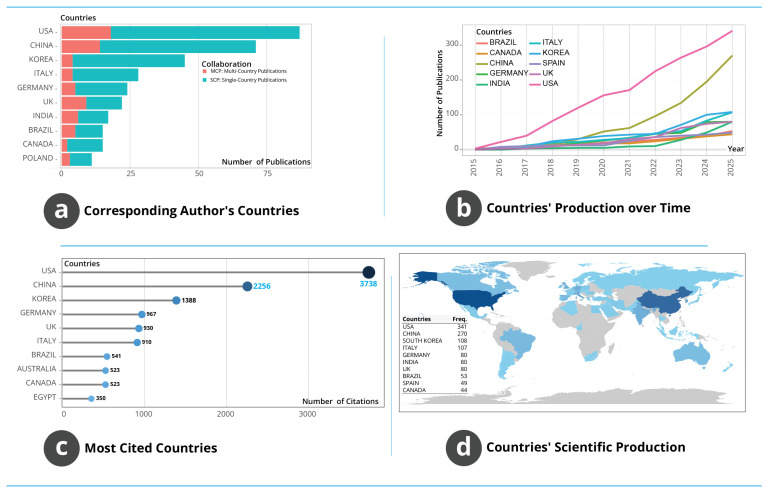
Geographical characteristics of AM–WT research. (**a**) Corresponding-author countries showing single-country and multi-country outputs. (**b**) National publication trajectories over time. (**c**) Countries ranked by local citation counts. (**d**) World map illustrating global distribution of publication activity.

The geographical structure of research activity, illustrated in Figure 8, shows clear concentration patterns shaped by national research capacity and cross-border collaboration. Figure 8a presents the distribution of corresponding-author countries and distinguishes between single-country and multi-country publications. The USA registers the highest output with more than 80 documents, followed by China and South Korea. Italy, Germany, the United Kingdom and India form a mid-range group, each producing roughly 20 to 35 publications. The relative proportions of single-country and multi-country publications indicate that the USA and China rely largely on domestic research ecosystems, whereas countries such as Germany and the United Kingdom exhibit stronger integration into international collaboration networks. Temporal trajectories in Figure 8b show that the USA and China follow the steepest and most sustained growth curves, particularly after 2019, surpassing 300 and 270 publications by 2025, respectively. South Korea and Italy also demonstrate substantial increases beginning around 2020. Countries including Germany, India, Brazil, Canada and Spain follow more gradual upward patterns, suggesting steady but less accelerated engagement. The inflexion points after 2020 across multiple countries indicate a period of intensified global research activity in AM and WT.

Figure 8c ranks countries by local citation counts and reveals differences between productivity and influence. The USA achieves the highest citation impact with 3738 citations, followed by China with 2256 and South Korea with 1388. Germany, the United Kingdom and Italy occupy intermediate positions with citation levels around 900 to 1000. These differences show that high output does not necessarily translate into proportional influence, and that several countries with moderate publication volumes demonstrate strong conceptual and methodological impact. The world map in Figure 8d visualises global publication density, highlighting the USA, China and South Korea as the dominant centres of activity. Italy, Germany, India and the United Kingdom appear as additional contributors with mid-level frequencies. Countries such as Brazil, Spain and Canada show visible but more limited engagement. The global distribution reflects a markedly uneven landscape in which research is concentrated in North America, East Asia, and parts of Europe, with minimal contributions from Africa, the Middle East, Latin America beyond Brazil and Oceania except for Australia. This pattern suggests that access to advanced manufacturing and microfluidic research infrastructure remains geographically unequal.

#### 3.1.9. High-Impact Publications and Foundational References

**Figure 9:**
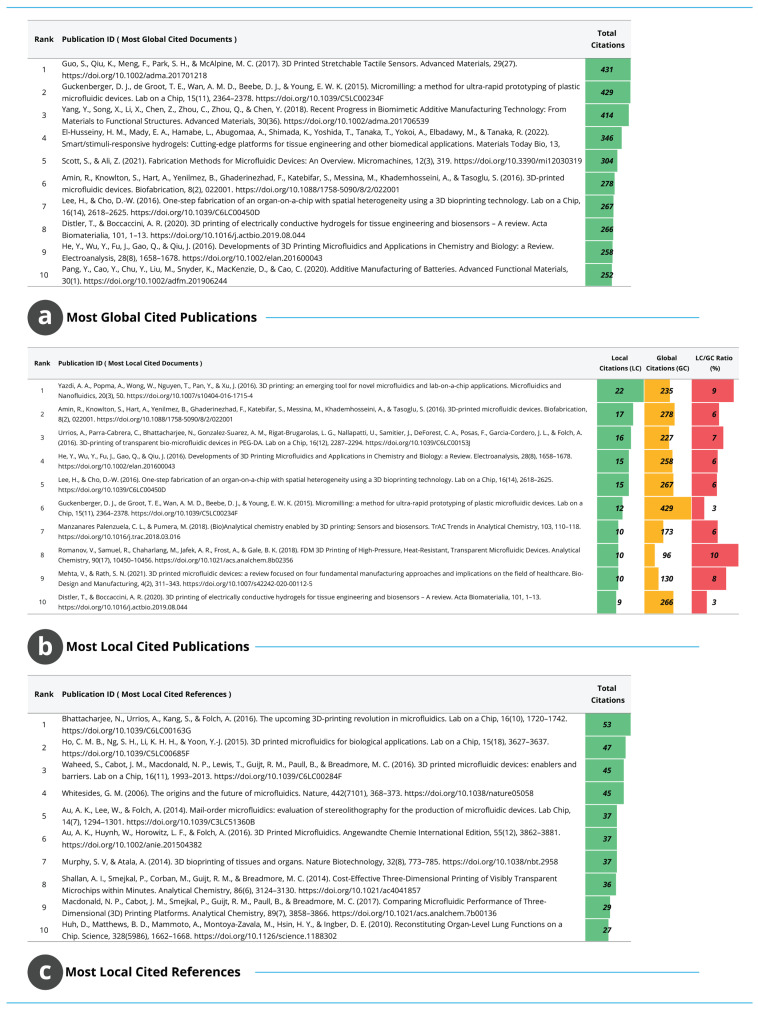
Citation structure of AM–WT research. (**a**) Most globally cited publications. (**b**) Most locally cited publications within the dataset. (**c**) Most locally cited foundational references informing contemporary research.

The citation structure illustrated in Figure 9 identifies the publications that have exerted the greatest influence within and beyond the field. Figure 9a ranks publications by global citation count, showing that the most widely recognised studies address tactile sensors, micromilling-based rapid prototyping and biomimetic AM, each attracting more than 400 citations. In addition, several highly influential papers, each exceeding 250 citations, concentrate on microfluidic device fabrication, organ-on-chip platforms, and stimuli-responsive hydrogel systems, underscoring the central role of these technologies within the field. These highly cited works indicate that enabling manufacturing methods, integrative microfluidic platforms and advanced functional materials form the conceptual and technological foundations, with reach extending across multiple research domains.

Figure 9b presents the most locally cited publications, capturing influence within the analysed corpus. Early contributions on 3D printed microfluidics, lab-on-a-chip systems and bioprinting-based device fabrication dominate this list, with local citation counts ranging from 15 to 22. The difference between global and local citation rankings shows that several studies with modest broader visibility hold central methodological importance within the AM–WT research space. These publications have become recurrent reference points for processes such as microfluidic device construction, organ-on-chip fabrication and rapid prototyping, which underpin the field’s technical development. Figure 9c lists the most locally cited foundational references, representing earlier literature that predates the dataset but continues to guide contemporary work. The highest-cited reference receives 53 local citations, with others clustered between 37 and 45 citations. These publications include seminal advances in stereolithographic manufacturing, transparent and flexible microfluidic systems, organ-level bioprinting and early demonstrations of 3D printed microfluidics. Their persistent citation indicates a stable reliance on a well-defined body of foundational knowledge that continues to inform fabrication strategies, microfluidic integration and device-level innovation. Overall, these three panels show that the field draws upon both recent high-impact contributions and longstanding foundational studies, forming a cumulative knowledge structure that integrates manufacturing technologies with biomedical and microfluidic applications.

#### 3.1.10. Topical Structure, Conceptual Focus and Thematic Evolution

**Figure 10:**
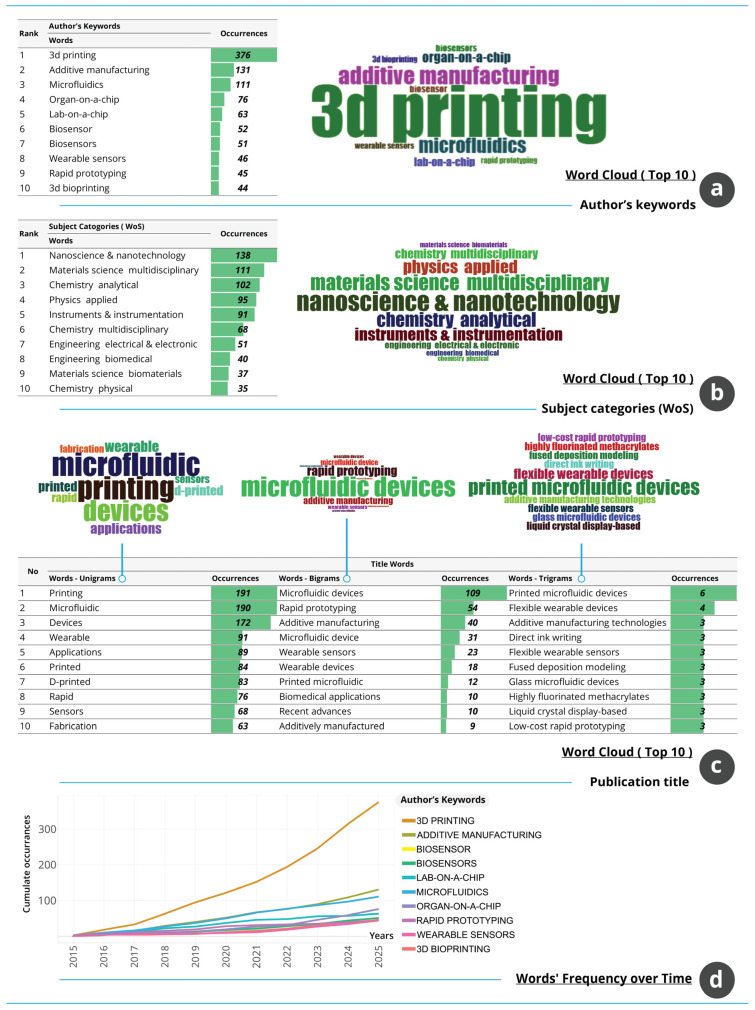
Topical structure and conceptual trends in AM–WT research. (**a**) Frequent Author’s Keywords and word cloud. (**b**) WoS subject-category distribution. (**c**) Most frequent title terms. (**d**) Temporal evolution of high-frequency keywords.

The topical and conceptual landscape of the field, illustrated in Figure 10, reflects a research domain structured around fabrication technologies, microfluidic systems and device-oriented applications. Figure 10a shows that the most frequent Author’s Keywords are dominated by 3D printing with 376 occurrences, followed by AM, microfluidics and application-focused terms such as organ-on-a-chip, lab-on-a-chip, biosensor and wearable sensors. This distribution indicates that the field is anchored in manufacturing processes while simultaneously engaging with device-level integration for biomedical and WT. Figure 10b presents the WoS subject categories and confirms the multidisciplinary positioning of the field. Nanoscience and nanotechnology, materials science, and analytical chemistry appear most frequently, supported by categories such as applied physics, instrumentation and biomedical engineering. These multidisciplinary patterns show that research activity is situated at the intersection of advanced materials design, microscale engineering and device-oriented biological interfaces.

Figure 10c analyses the most frequent title terms and demonstrates thematic coherence across unigrams, bigrams and trigrams. Terms such as printing, microfluidic, devices, wearable and applications reflect the primary conceptual areas, while combinations including microfluidic devices, rapid prototyping, AM and flexible wearable devices capture the operational relationships between fabrication methods and functional outputs. The recurrent emphasis on flexibility, micro-scale precision and device integration illustrates the continuity of technological priorities within the corpus. Figure 10d displays the temporal evolution of key terms. 3D printing shows the most pronounced increase over time, with marked acceleration after 2020. AM, microfluidics, biosensors and wearable sensors also display consistent upward trajectories, although at lower magnitudes. Application-specific terms demonstrate more moderate growth, indicating steady but focused expansion of niche subdomains. The collective upward trends suggest that the conceptual space is expanding rather than fragmenting, and that fabrication technologies remain central to the field’s evolving identity. Overall, the combined evidence demonstrates a coherent and expanding topical structure in which manufacturing processes, microfluidic platforms and wearable or biomedical applications form an integrated conceptual framework supported by strong multidisciplinary foundations.

#### 3.1.11. Conceptual Networks and Structural Connectivity

**Figure 11:**
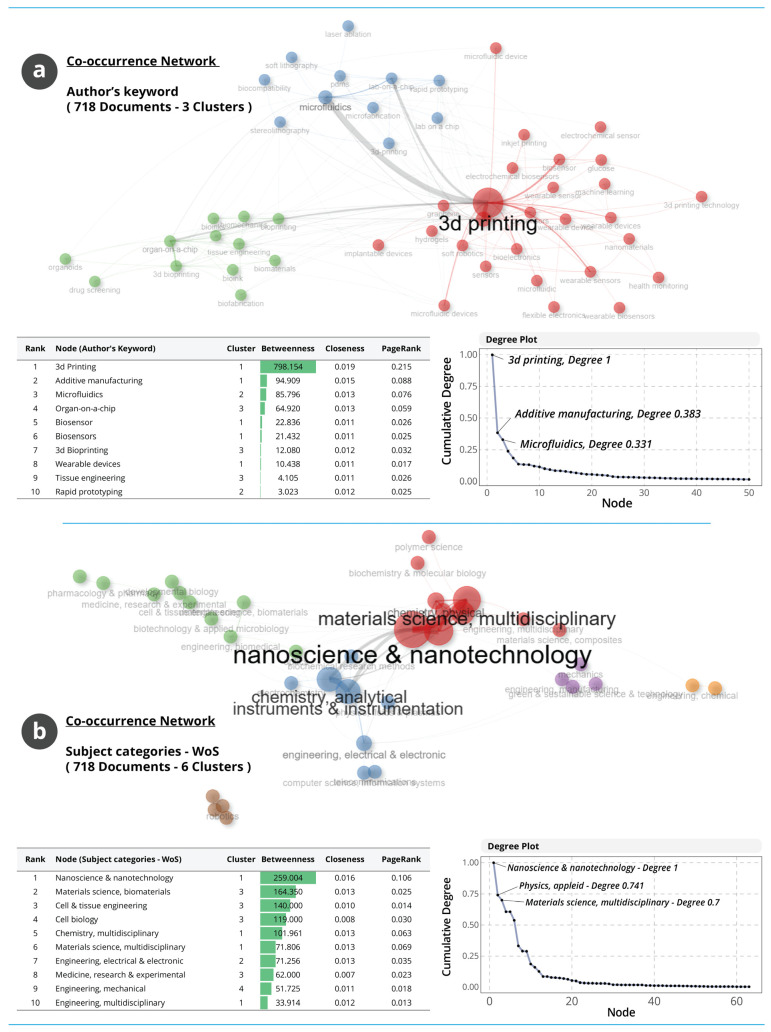
Conceptual co-occurrence networks in AM–WT research. (**a**) Author’s Keyword network showing cluster structure and centrality patterns. (**b**) Subject-category network illustrating disciplinary clustering and connectivity.

The conceptual networks in Figure 11 illustrate how key terms are organised within the field and how strongly they are interconnected through co-occurrence relationships. Figure 11a presents the co-occurrence network of Author’s Keywords and shows a structure dominated by 3D printing, which forms the central hub with the highest betweenness, closeness and PageRank values. AM and microfluidics serve as secondary hubs, linking fabrication technologies with device-oriented applications. The placement of terms such as organ-on-a-chip, biosensor, 3D bioprinting and wearable devices in intermediate positions indicates that applications in biomedical and wearable domains rely structurally on the conceptual pathways established by the dominant fabrication and microfluidic technologies. The steep centrality gradient, particularly the cumulative degree of 3D printing relative to other nodes, reflects a field with a strongly concentrated conceptual core.

Figure 11b shows the co-occurrence network of WoS subject categories and reveals six disciplinary clusters. Nanoscience and nanotechnology emerge as the highest-centrality category, followed by materials science, biomaterials and cell and tissue engineering. Categories such as chemistry multidisciplinary, materials science multidisciplinary and electrical and electronic engineering provide connective pathways between the physical sciences, materials design and integrated device technologies.

The degree distribution demonstrates that a small number of categories carry the majority of conceptual linkages, indicating a structurally coherent interdisciplinary architecture organised around nanoscale science, advanced materials and biomedical engineering. Overall, the networks depict a conceptual structure anchored in manufacturing and microfluidic technologies with strong integrative connections to biological and materials-focused domains. The prominence of central hubs and the steep decline in connectivity across nodes indicate a stable but highly focused intellectual ecosystem.

#### 3.1.12. Collaboration Networks and Structural Connectivity Across Authors and Countries

**Figure 12:**
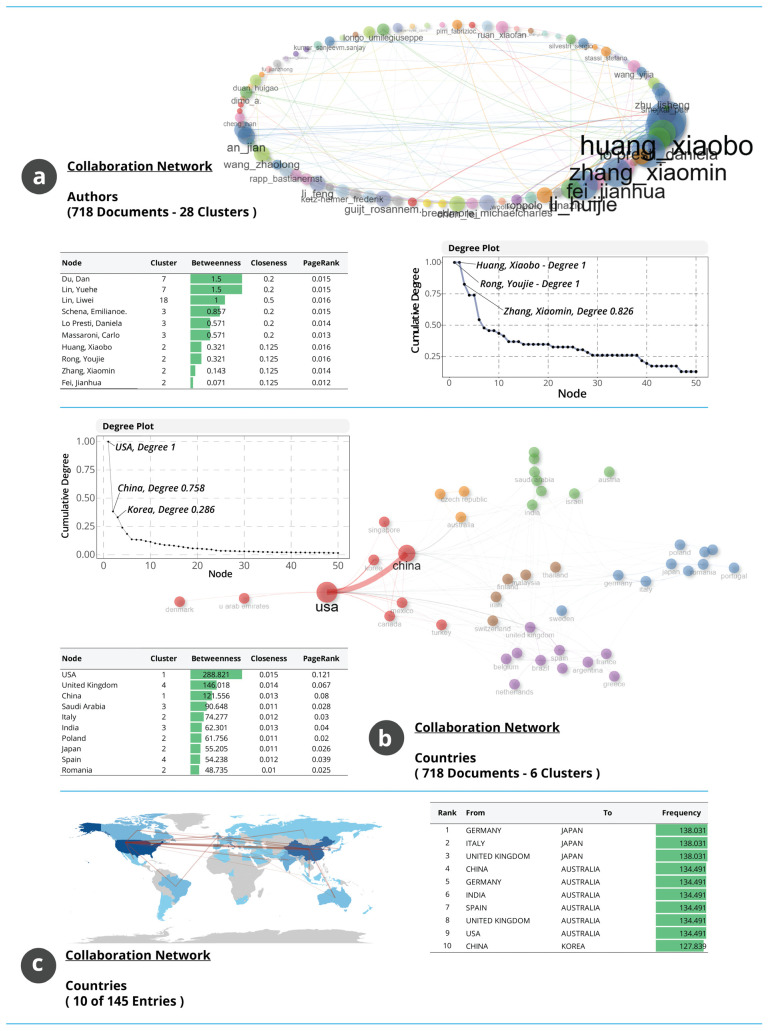
Collaboration networks in AM–WT research. (**a**) Author co-authorship network showing cluster structure and centrality patterns. (**b**) International collaboration network identifying major national hubs. (**c**) Country–country collaboration frequencies and global distribution of collaborative links.

The collaboration networks in Figure 12 characterise how research interactions are structured across authors and national systems. Figure 12a shows the author-level co-authorship network, comprising 28 clusters. The network is shaped by several densely connected groups and a small number of highly central individuals. Huang Xiaobo is the most influential connector with the highest cumulative degree, linking multiple clusters and facilitating knowledge exchange across otherwise weakly connected subgroups. Rong Youjie and Zhang Xiaomin also exhibit high connectivity, reflecting their participation in collaborations spanning different thematic areas. Authors such as Du Dan, Liu Yuehe and Lin Liwei have the highest betweenness values, indicating that they serve as structural bridges between research communities. The distribution suggests a collaboration ecosystem that relies on a limited number of influential authors to maintain cross-cluster cohesion. This structural dependency indicates that knowledge diffusion is mediated by a small number of bridging actors, which enhances thematic coherence but may simultaneously reduce epistemic diversity and slow the integration of peripheral innovation streams.

Figure 12b presents the international collaboration network, where the United States occupies the central position with the highest betweenness value, followed by the United Kingdom and China. These countries act as the primary conduits for cross-national knowledge flow. South Korea, Italy, India and Japan show moderate centrality, participating as regional hubs or thematic connectors. The degree distribution indicates a hierarchical structure in which a small number of national research systems support most international interactions. Figure 12c details directional country–country collaboration frequencies. The strongest links appear between Germany and Japan, Italy and Japan, and the United Kingdom and Japan, indicating Japan’s prominent role as a preferred collaborator across European partners. Additional strong links between China and Australia, Germany and Australia, and India and Australia show that Australia also functions as a major bilateral collaboration hub. The connection between China and Korea reflects strong regional integration within East Asia. The global map further highlights that the most active collaboration corridors occur between North America, East Asia, Western Europe and Australia, with limited participation from other regions. Overall, the networks show an uneven but highly interconnected collaborative structure in which a small number of influential authors and national systems anchor the majority of research interactions. These structural patterns facilitate thematic integration while reinforcing regional disparities in research capacity.

#### 3.1.13. Thematic Evolution and Structural Shifts in Research Focus

**Figure 13:**
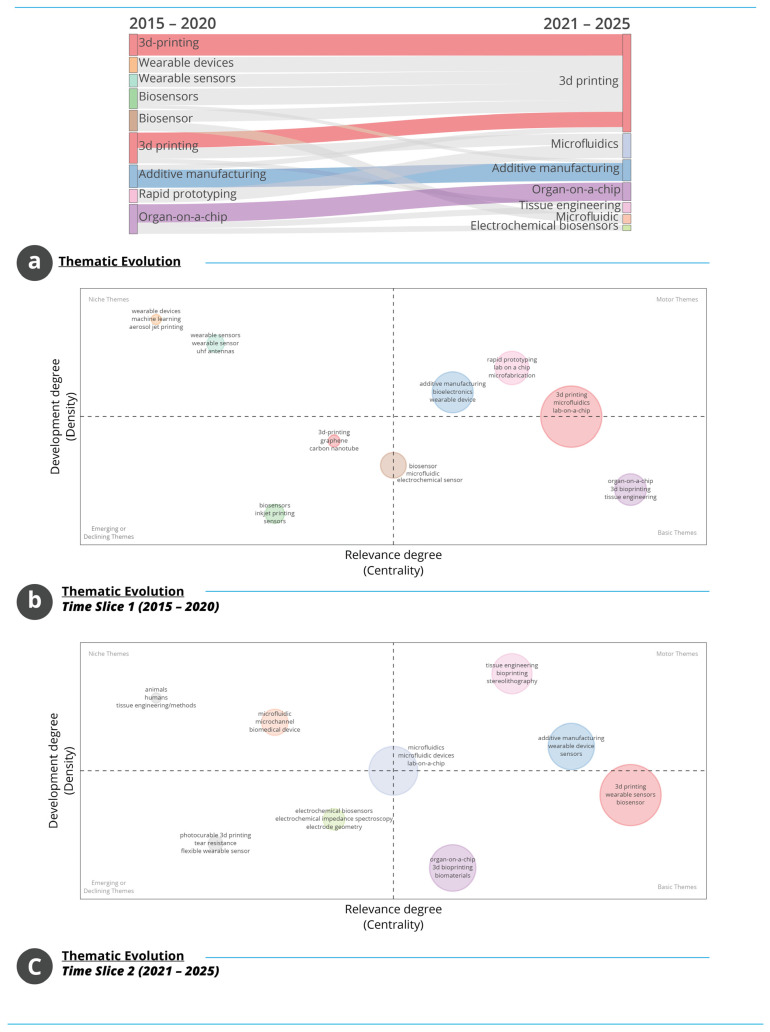
Thematic evolution in AM–WT research. (**a**) Transition of key themes between early and later periods. (**b**) Thematic map for 2015–2020 showing centrality–density positioning. (**c**) Thematic map for 2021–2025 illustrating shifts in thematic structure and consolidation.

The thematic structures in Figure 13 illustrate how the conceptual focus of the field has evolved between the early (2015–2020) and later (2021–2025) periods. Figure 13a shows that 3D printing remains the central and consistently expanding theme across both intervals. Terms such as wearable devices, wearable sensors, biosensors and rapid prototyping are prominent in the early period but increasingly converge towards broader frameworks such as AM and microfluidics in the later period. Themes including organ-on-a-chip and tissue engineering gain visibility in the second period, indicating a gradual shift towards biologically integrated fabrication approaches and more complex application domains.

Figure 13b presents the thematic map for 2015–2020 and shows a structure led by a strongly developed cluster composed of 3D printing, AM and lab-on-a-chip, positioned as a motor theme with high centrality and density. Rapid prototyping, microfabrication and bioelectronics also show elevated centrality, confirming their relevance within the period’s conceptual landscape. Conversely, themes such as wearable sensors, machine learning and aerosol jet printing occupy positions of higher density but lower centrality, suggesting specialised but less broadly connected research niches. Emerging or weaker themes, including biosensors and inkjet printing, appear in the lower-left quadrant, reflecting early-stage or declining emphasis. Figure 13c illustrates the thematic map for 2021–2025 and reveals substantial structural reconfiguration. A large basic-theme cluster composed of 3D printing, wearable sensors and biosensors emerges with high centrality but reduced density, indicating that these themes now underpin a broad range of studies. A second cluster combining AM, wearable devices and sensors displays strengthened density and centrality, reflecting convergence towards integrated device applications. Organ-on-a-chip, 3D bioprinting and biomaterials appear with moderate density and high centrality, suggesting developing but not yet saturated thematic areas. Terms such as electrochemical biosensors and microchannel occupy lower-density, lower-centrality positions, indicating emerging or peripheral activity. Compared with the earlier period, the thematic structure becomes more diverse, with increasing emphasis on biological integration and functional device development. The transition of ‘wearable sensors’ and ‘biosensors’ from peripheral or emerging positions in the 2015–2020 thematic map to central basic themes in 2021–2025 likely reflects a convergence of external technological and societal drivers. First, rapid advances in flexible electronics, multi-material additive manufacturing, and bio-compatible materials expanded the feasibility of structurally integrated sensing platforms. Second, the COVID-19 pandemic accelerated global demand for remote physiological monitoring and contactless diagnostic systems, reinforcing investment and publication activity in wearable health technologies. Third, influential publications and funding initiatives in bio-integrated devices and on-skin sensing platforms appear to have catalysed thematic consolidation, shifting these topics from exploratory niches toward foundational components of the AM–WT research architecture. Overall, the thematic evolution shows a field expanding from fabrication-centred foundations towards broader and more integrated technological applications. The transition between periods indicates increasing consolidation around manufacturing and microfluidic platforms, accompanied by the emergence of biologically oriented and application-driven research directions.

### 3.2. Comparative Discussion

#### 3.2.1. Bibliometric Objectives and Comparison with Previous Studies

Bibliometric analyses aim to synthesise large bodies of scientific knowledge by mapping publication trends, thematic dynamics, and collaboration structures. While prior reviews in AM or WT have offered valuable insights, many have remained limited to single-domain scopes or confined to Scopus-only data. For instance, de-la-Fuente-Robles et al. (2022) mapped WT-healthcare research using Scopus, while Romanelli et al. (2021) analysed AM trends via WoS [35,36]. Similarly, Cengiz Tirpan & Semiz (2022) conducted their analysis solely through Scopus, lacking multi-perspective triangulation [37]. The present study distinguishes itself by integrating records from WoS, Scopus, and PubMed, thereby achieving broader thematic saturation and source diversity. This cross-database design reduces coverage bias and captures both engineering- and biomedical-focused developments within AM–WT intersections. Previous studies, such as Dobrzańska-Danikiewicz & Bączyk (2024), conducted dual-database reviews in AM, but did not extend to WT or biomedical applications [38]. In contrast to domain-specific bibliometric studies that isolate either additive manufacturing or wearable technologies, the present analysis explicitly examines their intersection as an emergent convergence field, thereby capturing cross-domain thematic co-evolution rather than parallel disciplinary trajectories. Thus, by merging three distinct databases, this study offers a more holistic and structurally connected view of how AM and WT research evolve concurrently, particularly where fabrication techniques and bio-integration intersect. Unlike prior studies that relied exclusively on a single database (e.g., Scopus or WoS), the present work integrates three complementary sources and applies DOI-level deduplication combined with full-name author disambiguation, thereby reducing homonym bias and database coverage asymmetry. Furthermore, while earlier analyses primarily emphasised publication counts and keyword frequency, this study incorporates logistic growth modelling, centrality-based network diagnostics, and thematic life-cycle mapping to examine structural maturity and knowledge diffusion. This triangulated approach supports more broadly applicable and cross-disciplinary insights without overstating novelty. In doing so, the study aligns with recommended bibliometric procedures, including multi-source integration, clear inclusion criteria, and structured thematic mapping [24,39]. These methodological standards ensure that the outcomes are not only representative but also analytically robust, supporting both retrospective evaluation and prospective trend identification. Consequently, the analysis meets the core objectives of bibliometric research as outlined in systematic review frameworks [40].

#### 3.2.2. The Present Study Highlights Key Scientific Trends

This study reveals a significant rise in research intersecting AM and WT since 2015, peaking sharply after 2020, paralleling trends noted in sector-specific reviews [41,42]. Citation patterns show disproportionately high impact for studies involving biomedical sensors and soft robotics, consistent with broader interest in healthcare-aligned AM-WT integration [37]. Institutional outputs highlight a concentration of productivity in the United States and China, yet regional citation influence appears more dispersed, a finding also reported in WT-related cardiovascular research [43]. Compared to previous domain-specific studies, this work offers a longitudinal and cross-disciplinary perspective, enabling a more nuanced understanding of how fabrication technologies support emerging bio-integrated applications. By linking thematic structures with performance indicators across databases, the study uncovers convergence patterns not clearly identified in single-domain analyses.

#### 3.2.3. Emerging Trends and Thematic Shifts in AM Connected WT Research

In the past decade, research at the intersection of AM and WT has undergone a marked thematic transformation. Early studies focused primarily on the engineering aspects of AM, such as 3D printing, material design, and mechanical testing, with minimal attention to integration with wearables. Our co-word and overlay analyses (Figure 8, Figure 9 and Figure 10) demonstrate that post-2018, a clear trend emerged towards health-focused and human-integrated applications, including biosensing, flexible electronics, skin-conformal devices, and soft robotics. This trajectory reflects a shift from enabling technologies to real-world functionality. Keywords such as “bio-integrated,” “non-invasive,” and “real-time monitoring” exhibit high centrality and temporal emergence, signalling their rising importance within AM-WT research. This thematic shift mirrors similar trends previously reported in the literature, including growing interest in hybrid fabrication methods for soft, bio-compatible electronics [44], and increasing diversification in AM outputs [41]. Unlike earlier reviews that treated AM and WT as separate or sequential fields, our analysis illustrates their growing co-dependence, especially in healthcare, sports, and human–machine interface applications. The convergence suggests not only maturing fabrication techniques but also evolving priorities in user-centred, adaptive technologies.

Additionally, the bibliometric trajectory indicates an impending evolution of WT–AM research toward higher convergence with biomedical customisation, flexible energy systems, and autonomous sensing. Keywords such as “organ-on-chip”, “self-powered sensors”, and “bioprinting” signal nascent trends that may define the field’s next phase. These insights are not merely descriptive but serve as foresight instruments to anticipate where funding, collaboration, and theoretical efforts should pivot.

#### 3.2.4. Structural Gaps and Underexplored Domains

The present analysis uncovers notable structural imbalances in AM–WT research. First, a significant concentration of output is visible in a limited set of institutions and countries, primarily the United States, China, and select Western European nations. While these actors dominate co-authorship and citation networks, contributions from Africa, South America, and Southeast Asia remain sporadic and peripheral, echoing global disparities reported by Obi et al. (2022) [45]. Second, thematic saturation in areas such as biomedical sensors and 3D-printed prosthetics contrasts with a limited exploration of wearable environmental monitors, smart textiles, and non-invasive drug delivery, suggesting potential for diversification. Additionally, our analysis highlights a methodological narrowness: most studies favour co-authorship and co-word techniques, while advanced frameworks such as temporal path analysis or citation context modelling remain rarely applied. These patterns point to both opportunity and oversight. Without broader institutional participation and methodological plurality, innovation risks being funnelled through existing dominant clusters. Similar regional and thematic gaps have been observed in bibliometric studies of WT by Kageyama et al. (2022), reinforcing the need for more inclusive and exploratory research agendas [43].

#### 3.2.5. Future Directions for AM-Connected WT Studies

This study underlined that the convergence of AM and WT is expected to pivot towards more integrative applications such as personalised health monitoring, multi-material bioprinting, and smart wearable systems. Emerging topics include AI-enhanced wearables, soft robotics, and hybrid manufacturing pathways. However, as highlighted in prior reviews, domains like non-invasive diagnostics and environmental sensing remain underexplored [46,47]. Future research should address these gaps by adopting interdisciplinary frameworks and leveraging real-time data integration across biosensors, materials science, computational modelling, and artificial intelligence to drive innovation.

#### 3.2.6. Actionable Implications for Stakeholders

Based on the observed thematic gaps and collaboration asymmetries, funding agencies may prioritise programmes that (i) support cross-disciplinary consortia spanning additive manufacturing, microfluidics, flexible electronics and clinical translation, and (ii) target underrepresented application spaces including smart textiles, environmental wearables and sustainability-oriented material/process development for wearable devices. Universities can operationalise these priorities by fostering joint laboratories and co-supervision models between manufacturing engineers, materials scientists, bioengineers and health researchers, thereby reducing structural reliance on a small number of national hubs. For researchers entering the field, the most promising opportunities indicated by the present mapping include smart-textile integration, wearable environmental monitoring, and sustainable AM routes for compliant, skin-conformal devices, where thematic density remains comparatively limited despite high application relevance.

### 3.3. Study-Specific Limitations and Prospective Avenues for Future Reviews

This study aimed to systematically map the intellectual landscape at the intersection of AM and WT. While it employed a multi-database approach (WoS, Scopus, PubMed) and integrated validated protocols for disambiguation and thematic mapping, several limitations persist. One core limitation lies in the dependency on bibliographic metadata, which constrains deeper semantic analysis and limits the interpretability of intra-cluster conceptual depth, a frequently cited challenge in bibliometric studies [48]. In particular, keyword co-occurrence captures topical salience but cannot distinguish between studies that merely reference a given technology and those that provide substantive methodological or performance advances, which may inflate the apparent maturity of some thematic clusters. To mitigate this, we implemented full author name disambiguation using RStudio-based scripts, manually verifying institutional affiliations and co-authorship patterns. This strategy aimed to resolve inconsistencies caused by truncated or duplicated author entries across databases, thereby improving author-level accuracy and network coherence. Despite this refinement, disparities in database indexing policies and keyword taxonomies may still have led to partial thematic overlaps or skew, even after harmonisation. The exclusion of non-English and grey literature introduces representational bias, potentially underestimating contributions from emerging research regions [49]. The exclusion of abstract-level searching may have reduced corpus breadth; however, this constraint was applied to preserve thematic specificity and minimise peripheral inclusion bias. Further, thematic structuring relied primarily on keyword co-occurrence and clustering logic, rather than ontology-driven classification, which may limit conceptual fidelity in highly interdisciplinary domains [50]. Nevertheless, the applied methodological pipeline adheres to robust bibliometric protocols and enables a reliable base for cross-domain trend exploration. Future reviews may enhance thematic granularity through hybrid designs integrating content analysis, NLP-based citation contexts, and longitudinal topic modelling. Cross-validating bibliometric trends with experimental or market data may also deepen insights, particularly as AM–WT applications evolve toward personalised healthcare, adaptive interfaces, and sustainable material cycles [51].

## 4. Conclusions

This study provides a comprehensive bibliometric mapping of research at the intersection of AM and WT, addressing a notable gap in the previous literature, where these domains were typically analysed in isolation. By examining the convergence itself as a structured knowledge domain and integrating three complementary databases with enhanced disambiguation procedures, the present synthesis reveals structural patterns and thematic transitions that cannot be inferred from additive manufacturing or wearable-technology analyses conducted independently. By merging datasets from WoS, Scopus, and PubMed, and applying validated disambiguation and thematic mapping protocols, this work constructs a unified knowledge structure revealing both historical trajectories and future directions in AM–WT convergence.

Key findings indicate a thematic shift from production-centric research, dominated by fabrication processes and mechanical design, toward user-centred applications such as biosensing, soft robotics, and skin-integrated systems. Network analyses highlight structural asymmetries in global collaboration, with overrepresentation from a few research clusters and underexplored regions. Despite these disparities, a progressive co-dependence between AM and WT is evident, especially in health monitoring and adaptive systems.

The study also identifies methodological blind spots in the literature, such as limited multi-database integration and thematic scope, which it addresses through enhanced metadata processing and comparative evolution analysis. However, limitations remain in semantic granularity and grey literature coverage. The framework and insights offered here serve as both a roadmap for emerging trends and a foundation for future bibliometric and content-based hybrid reviews.

This work not only clarifies the current intellectual structure of AM–WT research but also underscores strategic gaps and latent opportunities, fostering a deeper understanding of how fabrication technologies and wearable innovations co-evolve in response to societal and technological demands.

## Figures and Tables

**Table 1:** Overview of the bibliometric analytical framework, tools, and corresponding outputs.

Analytical Phase	Objective	Tool/Procedure	Key Metrics/Models	Corresponding Tables/Figures
Data Collection	Retrieve AM–WT publications	WoS, Scopus, PubMed	Boolean query (title + keywords)	Figure 1
Data Integration	Merge and clean datasets	convert2df() + custom R scripts	DOI-based deduplication; metadata completeness scoring	Figure 1
Author Disambiguation	Resolve homonym ambiguity	Full-name tokenisation (AF field)	Unique author tokens	Figures 6 and 12
Descriptive Analysis	Characterise corpus structure	Bibliometrix	Growth rate, citation averages, document types	Table 2, Figure 2
Growth Modelling	Assess life-cycle stage	Logistic regression modelling	R^2^, projected peak, saturation level	Figure 4
Source Analysis	Identify core journals	Bradford’s Law; H-index	Source productivity and local citation impact	Figure 5
Author and Institution Analysis	Evaluate productivity and influence	Lotka’s Law; local citations	Publication counts; centrality	Figures 6 and 7
Geographic and Collaboration Analysis	Map international structure	Network centrality metrics	Betweenness, degree	Figures 8 and 12
Thematic Mapping	Identify conceptual structure	Co-word analysis; thematic maps	Centrality–density quadrants	Figures 10 and 13
Conceptual Network Analysis	Examine structural connectivity	Co-occurrence networks	Betweenness, closeness, PageRank	Figure 11
Citation Structure	Identify foundational works	Global and local citation analysis	Citation counts	Figure 9

**Table 2:** Descriptive statistics for the analysed publication corpus.

Description	Results
**Main Information about Data**	
Timespan	10 October 2015–10 October 2025
Sources (Journals, Proceedings, etc.)	315
Documents (Publications)	718
Annual Growth Rate %	24.89
Document Average Age (year)	4.52
Average Citations per Doc.	29.33
References	29,275
**Document Contents**	
Keywords Plus (ID)	3854
Author’s Keywords (DE)	1971
**Authors**	
Authors	3496
Authors of Single-authored Docs (Number of unique authors)	11
**Authors Collaborations**	
Single-authored Docs (Total number of single-authored docs)	12
Co-Authors per Doc.	5.66
International Co-authorships %	15.46
**Documents Type**	
Journal Article	420
Conference/Proceedings Article	131
Review Article	167

## Data Availability

The dataset underpinning this study is available from the authors upon reasonable request.

## Abbreviations

The following abbreviations are used in this manuscript:

3D	Three Dimensional
4D	Four Dimensional
AM	Additive Manufacturing
DOI	Digital Object Identifier
PRISMA-ScR	Preferred Reporting Items for Systematic Reviews and Meta-Analyses for Scoping Reviews
WoS	Web of Science
WT	Wearable Technology
